# Effects of sintering temperature on sensing properties of WO_3_ and Ag-WO_3_ electrode for NO_2_ sensor

**DOI:** 10.1098/rsos.171691

**Published:** 2018-10-31

**Authors:** Rui Lu, Xiaoling Zhong, Shiguang Shang, Shan Wang, Manling Tang

**Affiliations:** 1 College of Information Science and Technology, Chengdu University of Technology, Chengdu 610059, People's Republic of China; 2 School of Electronic Engineering, Xi'an University of Posts and Telecommunications, Xi'an 710121, People's Republic of China; 3 Faculty of Automation and Information Engineering, Xi'an University of Technology, Xi'an 710048, People's Republic of China

**Keywords:** tungsten trioxide, gas sensing properties, sintering temperature, low operating temperature

## Abstract

Pure WO_3_ and Ag-WO_3_ (mixed solid solutions Ag with WO_3_) have been successfully synthesized by sol-gel method and the influences of calcination temperature on the particle size, morphology of the WO_3_ and Ag-WO_3_ nanoparticles were investigated. Powder X-ray diffraction results show that the hexagonal to monoclinic phase transition occurs at calcination temperature varying from 300°C to 500°C. SEM images show that calcination temperature plays an important role in controlling the particle size and morphology of the as-prepared WO_3_ and Ag-WO_3_ nanoparticles. The NO_2_ gas sensing properties of the sensors based on WO_3_ and Ag-WO_3_ nanoparticles calcined at different temperatures were investigated and the experimental results exhibit that the gas sensing properties of the Ag-WO_3_ sensors were superior to those of the pure WO_3_. Especially, the sensor based on Ag-WO_3_ calcined at 500°C possessed larger response, better selectivity, faster response/recovery and better longer-term stability to NO_2_ than the others at relatively low operating temperature (150°C).

## 1. Introduction

Metal oxides have been very important materials in catalysis, gas sensors and energy conversions [1]. Tungsten trioxide (WO_3_), a typical n-type semiconductor material with a band gap of 2.5–2.8 eV, has received much interest for applications including photocatalysis [2–5], electrochromic devices [6–8], solar energy conversion [9] and gas sensors [10], due to excellent catalytic, optical and dielectric properties, good physical and chemical stability. For gas sensing applications, WO_3_ has attracted great attention for its distinctive sensing properties, and has been regarded as a promising material for detecting various gases, including CO [11], H_2_ [12–13], SO_2_ [14], NO*_χ_* [15–19], H_2_S [20] and organic vapours [21]. In addition, tungsten trioxide (WO_3_) has been considered as a promising sensing material of solid-state semiconductor gas sensors for NO_2_ monitoring because of its excellent sensitivity and selectivity. WO_3_ nanoparticles can be fabricated by various techniques such as chemical vapour deposition [22], hydrothermal method [23–26], microwave irradiation method [27] and sol-gel process [28]. Sol-gel technique is the most common method of fabricating WO_3_ nanoparticles because of the advantages of simple, quick process and easy control of particle size, crystal structure and morphology. In recent years, many attempts have been made to enhance the gas sensitivity of semiconductor gas sensors [29], one of which involved the doping of noble metal in the materials. It has been shown that the sensing performance of WO_3_ can be substantially improved by loading particular elements [30–33]. Wang *et al*. [34] have shown that a high sensitivity was achieved when noble metals such as Pt, Au and Pd were deposited as activator layers on WO_3_ films. Najim *et al*. [35] have mixed SnO_2_ with WO_3_ and prepared to synthesize nanostructured thin films by pulsed laser deposition as gas sensor. The sensors showed high sensitivity. However, there is still room for improvement in stability, selectivity and working temperature of the gas sensors [36].

In this work, WO_3_ nanoparticles and Ag-WO_3_ were prepared by a simple sol-gel method. The influences of calcination temperature on the particle size, morphology of the WO_3_ and Ag-WO_3_ nanoparticles were extensively investigated and NO_2_ gas sensing properties of WO_3_ and Ag-WO_3_ nanoparticles were discussed.

## 2. Material and methods

### 2.1. Preparation and characterization

The WO_3_ and Ag-WO_3_ nanoparticles were prepared by sol-gel method. All of the chemical reagents were of analytical grade and used as received without further purification. In a typical synthesis, 3.5 g tungsten (W) powder was dissolved into 200 ml of deionized water under constant stirring for 30 min. Then, 100 ml of hydrogen peroxide (H_2_O_2_) were added into the above solution and stirred for 20 min. A homogeneous WO_3_ precursor solution was produced by slowly dropping 30 ml of alcohol and stirred in the thermostatic water bath at 80°C until an opaque gel was formed. Ag-WO_3_ precursor solution was also fabricated by dropping 4 ml of 0.1 mol l^−1^ AgNO_3_ aqueous solution into the WO_3_ precursor solution. The pH value of the solution was fixed at 4.5 which was adjusted by using nitric acid (HNO_3_) solution in the reaction process. After that, the transparent gel was transferred into a crucible and baked at 80°C for 12 h. Finally, the obtained products were calcined at 300°C, 500°C and 700°C for 2 h. A series of pure WO_3_ and Ag-WO_3_ powders were obtained.

### 2.2. Fabrication and measurement of gas sensor

The products’ crystallographic structures information were observed with small angle X-ray diffraction in Shimadzu diffractometer (XRD-6000, Japan) using Cu Kα line radiation at 40 kV and 40 mA. The XRD patterns were collected at 2 angles of 10–80° at a scan rate of 1° min^−1^. A field emission scanning electron microscope (FESEM, JSM-6700F, Japan) was used to measure the particle size and morphology of WO_3_ nanoparticles. Brunauer–Emmett–Teller (BET) method was used to determine the pore size distributions and surface areas. The gas sensing property of Ag-WO_3_ nanoparticle film was measured by semiconductor characterization system (Keithley, 4200-SCS) with interdigital electrode structures. The concentration of Ag and W were measured by inductively coupled plasma–atomic emission spectroscopy (ICP-AES, Vista).

**Figure 1.**
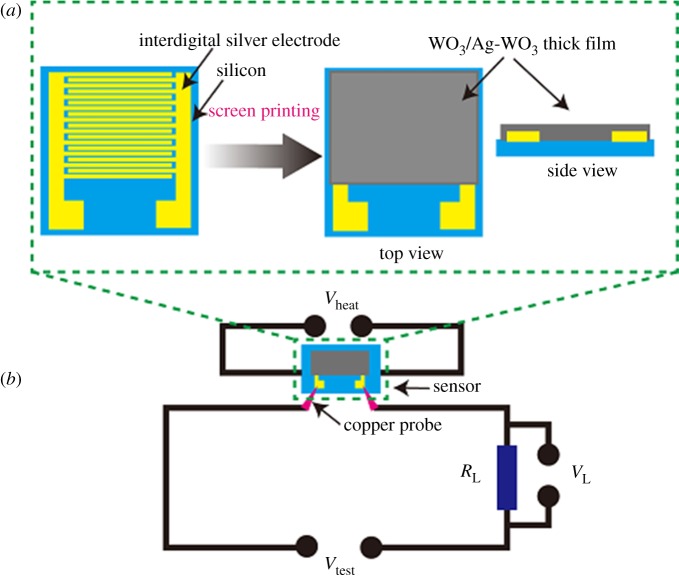
(*a*) Structure of thick film sensor and (*b*) measuring electric circuit of gas sensing properties.

Side-heating gas sensors were built to measure the gas sensing properties of WO_3_ and Ag-WO_3_-based nanoparticles. Figure 1*a* shows the structure of thick film sensor, a commercial Si substrate with dimensions of 15 × 9 × 0.76 mm, and built-in Ag electrodes at 0.5 mm intervals. The WO_3_ and Ag-WO_3_ nanoparticles paste were coated in a 35 µm thickness via the screen-printing method and then sintered at 300°C for 15 days in air, in order to improve their stability and repeatability. The measuring electric circuit of gas sensing properties is shown in figure 1*b*. The operating voltage (*V*_heat_) was supplied to heat the sensor with A Ni-Cr heater, which can control the operating temperature from 100 to 500°C, and a test voltage (*V*_test_) was supplied across. A load resistor *R*_L_ was connected in series with the sensor, that resistance was measured and used for calculating and outputting the corresponding sensor resistance.

**Figure 2.**
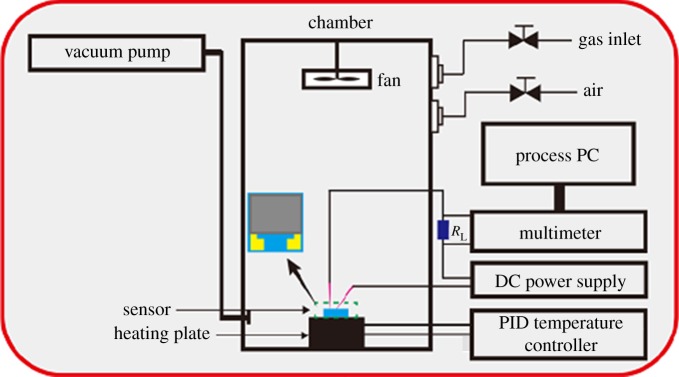
Apparatus used for gas sensing experiments.

A sensor performance testing apparatus is shown in figure 2. The sensor was installed at a distance of 70 mm from the bottom of a 20 l (500 × 200 × 200 mm) chamber. The sensing electrodes were connected to the test circuit by copper probes. After the target gas was injected into the chamber with the fan on, the resistance was measured with an electrometer after the equilibrium concentration was reached. The sensitivity (*S*) of the sensing electrodes was defined as: *S* = *R*_a_/*R*_g_ for reducing gases or *S* = *R*_g_/*R*_a_ for oxidizing gases, where *R*_a_ and *R*_g_ represent the resistances of the sensing electrodes in air and in a target gas, respectively. Furthermore, the response and recovery times were defined as the times at which a total resistance change of 90% was achieved.

## 3. Results and discussion

### 3.1. X-ray diffraction

**Figure 3.**
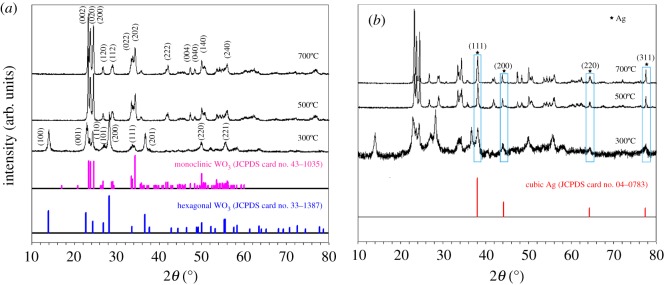
XRD diffraction patterns of (*a*) pure WO_3_ and (*b*) Ag-WO_3_ nanoparticles at different calcination temperatures.

Figure 3 shows X-ray diffraction (XRD) patterns of (*a*) pure WO_3_ and (*b*) Ag-WO_3_ nanoparticle heated at 300°C, 500°C and 700°C for 2 h. As shown in figure 3*a*, the observed peaks at 300°C could be well matched to the pure hexagonal phase of WO_3_, and agree well with the values in the standard card (JCPDS 33–1387). The main characteristic diffraction peaks at 500°C and 700°C correspond to the (002), (020), (200) and (202) planes at 2*θ* = 23.12^o^, 23.59^o^, 24.38^o^ and 34.16^o^, respectively. These peaks match perfectly with the monoclinic structure of WO_3_ according to the JCPDS file card (JCPDS 43-1035). Furthermore, the width of the peak at 700°C is sharper than that at 500°C. It indicates that the crystallite size of 700°C increases with temperature. No peaks of impurities can be found in XRD patterns of pure WO_3_, illustrating the as-prepared samples were of high purity. The XRD patterns of Ag-WO_3_ nanoparticles at different calcination temperatures are reported in figure 3*b*. All peaks are well matched with the diffraction lines documented for the hexagonal tungsten oxide (JCPDS 33-1387), monoclinic tungsten oxide (JCPDS 43-1035) and cubic Ag (JCPDS 04-0783). The diffraction peaks of Ag (111), (200), (220) and (311) at 38.1°, 44.0°, 64.5° and 77.5° can be observed obviously. The effect of the calcination temperature and mixing Ag on the crystallite dimensions of WO_3_ was also detected by XRD. The average crystal size of WO_3_ was estimated by using the Scherrer equation:

$$D=\frac{\kappa \lambda }{\beta \cos\theta },$$

where *D* is the crystalline size, *κ* is the so-called shape factor and usually taken as 0.89, *λ* and *θ* are the radiation wavelength (0.154056 nm) and Bragg's angle, respectively, *β* is the full width at half maximum (FWHM) of the diffraction peak. The average grain sizes of pure WO_3_ and Ag-WO_3_ particles calcined at 300°C, 500°C and 700°C were about 235, 343 and 414 nm, 98, 129 and 314 nm, respectively. To measure the precise amount of Ag in the Ag-WO_3_, ICP-AES was used. The results show that the mass percentage of Ag in Ag-WO_3_ is 0.9 wt%. Indicating that the grain size of as-prepared WO_3_ grew with increasing calcination temperature. Moreover, the average grain sizes decreased slightly by mixing Ag, which may be due to a small amount of Ag loaded in the mesoporous WO_3_ or a homogeneous distribution of Ag particles [37].

### 3.2. Field emission scanning electron microscopy

**Figure 4.**
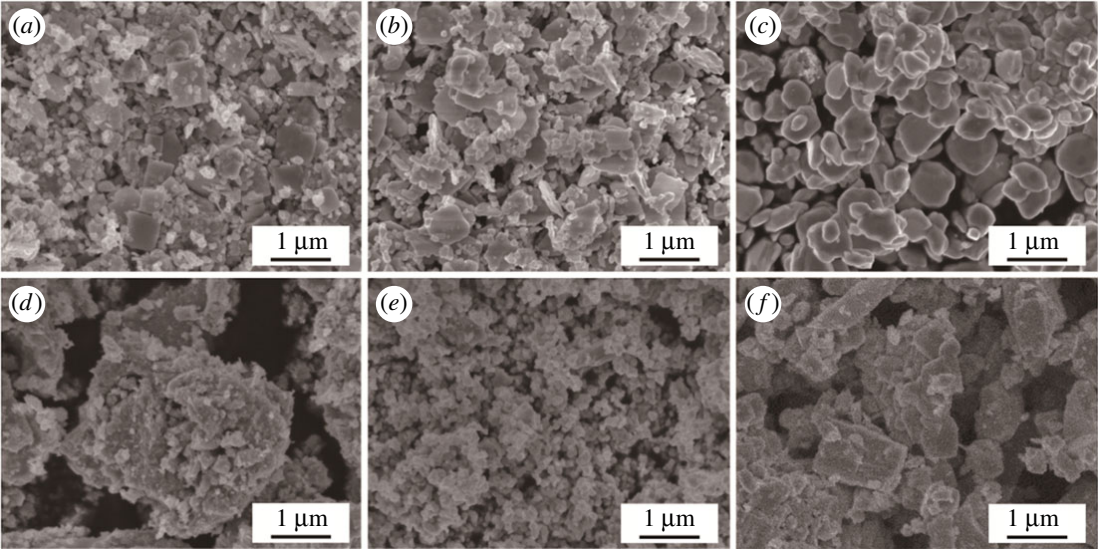
SEM images of WO_3_ (*a–c*) and Ag-WO_3_ (*d–f*) nanoparticles at different calcination temperatures (*a,d*) 300°C, (*b,e*) 500°C and (*c,f*) 700°C.

Figure 4 shows the SEM micrographs of pure WO_3_ (*a*–*c*) and Ag-WO_3_ (*d*–*f*) nanoparticles samples heated at 300°C, 500^o^C and 700°C, respectively. As illustrated in figure 4*a–c* the irregular mixture of sliced or granular structure at 300°C and 500°C, and mainly spherical particles of diameter 50–800 nm with irregular fringe are observed. When the calcination temperature increases to 700°C as shown in figure 4*c*, the powder presents three-dimensional (3D) irregular microspheres and some of them are interconnected with each other. Furthermore, larger size particles appear and tend to be of irregular shape with straight edges, which can be attributed to the thermally promoted crystallite growth. Figure 4*d–f* shows the SEM images of Ag-WO_3_ at different calcination temperatures. The images of the Ag-WO_3_ powder calcined at 300°C is shown in figure 4*d*. Ag-WO_3_ particles tend to form large agglomerates due to physical attraction between the particles with small sizes and irregular shapes. As the temperatures raise to 500°C, figure 4*e*, the particles showed good homogeneity and discreteness. Figure 4*f* shows the irregular mixture of sliced or granular structure with significant agglomeration at 700°C. The experiment results indicate that Ag-WO_3_ of the sol-gel system for the fabrication of uniform nanoparticles of hexagonal and monoclinic WO_3_ from condensed WO_3_ gel is a key factor for controlling the final particle size and shape of the product.

**Figure 5.**
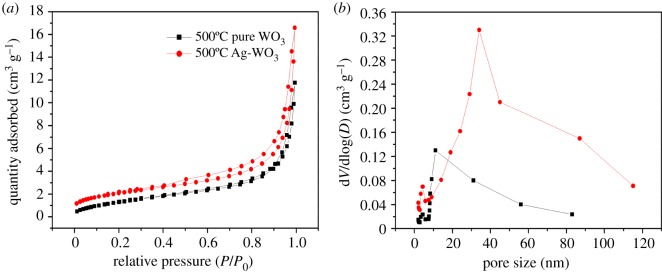
(*a*) Nitrogen adsorption–desorption isotherms and (*b*) pore diameter distribution curves of WO_3_ and Ag-WO_3_ calcined at 500°C.

Nitrogen gas sorption analyses were carried out to study the porosity of the composites. Figure 5*a* shows nitrogen adsorption–desorption isotherms and figure 5*b* pore diameter distribution curves of WO_3_ and Ag-WO_3_ calcined at 500°C. More details about nitrogen adsorption–desorption isotherms and pore diameter distribution curves can be found in the electronic supplementary material.

### 3.3. Gas sensing characteristics

Both of the nitrogen adsorption–desorption isotherms are type IV curves, characteristic of mesoporous materials [38,39]. The average values of the specific surface area and pore sizes calculated by the Barrett–Joyner–Halenda (BJH) method are illustrated in table 1. The specific surface area of the pure WO_3_ powders decreased sharply with the increasing sintering temperature more than 500°C due to the grain sintering, phase transformation and growing up. The pore size of Ag-WO_3_ is larger than the diameter of the WO_3._ The surface area increases slightly by mixing Ag at the same temperature, which may be due to the incorporation of Ag attached to the WO_3_ attached to the WO_3_ framework affects the integrity and mesostructure [40]. Futuremore, the biggest specific surface area and pore size can be obtained with Ag-WO_3_ calcined at 500°C.

**Table 1.** Physical properties of the WO_3_ and Ag-WO_3_ calcined at different temperatures.

sintering temperature (°C)	BET specific surface area (m^2^ g^−1^)	pore size (nm)
300°C (pure WO_3_)	5.4	10.5
500°C (pure WO_3_)	4.7	9.7
700°C (pure WO_3_)	4.4	7.6
300°C (Ag-WO_3_)	5.9	24.7
500°C (Ag-WO_3_)	6.7	32.6
700°C (Ag-WO_3_)	4.8	14.5

**Figure 6.**
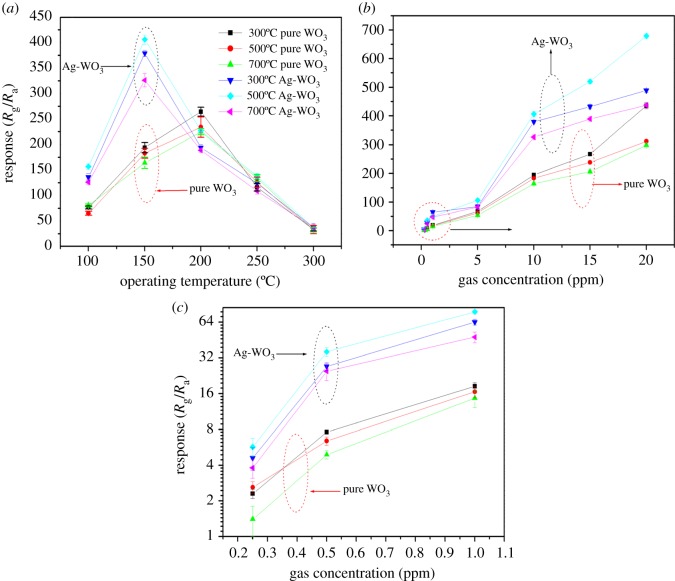
(*a*) Gas sensing response of the sensors based on pure WO_3_ and Ag-WO_3_ at different calcined temperatures to 10 ppm NO_2_. (*b,c*) Responses of pure WO_3_ and Ag-WO_3_ sensors to different concentration NO_2_ at 150°C.

The gas sensing properties of WO_3_ and Ag-WO_3_ at different calcination temperatures to 10 ppm NO_2_ were measured at various operating temperatures, as shown in figure 6*a*. It is obvious that the response of these sensors to 10 ppm NO_2_ varies with not only the operating temperature but also mixing Ag. It can be seen that all the sensitivity change shows a sharp upward trend at first and decreased rapidly with an increase in operating temperature. For all the Ag-WO_3_ sensors, there is a maximum value at 150°C, while all the pure WO_3_ sensors have the maximum gas response at 200°C. The operating working temperature of all the Ag-WO_3_ sensors is lower than pure WO_3_ sensors. In addition, the sensitivity of Ag-WO_3_ sensors exhibits much higher response than the pure WO_3_ sensors, compared with previous reports about NO_2_ sensors [41–43]. Especially, the Ag-WO_3_ sensor calcined at 500°C presents the largest response to NO_2_ at 150°C, which indicates that the sensitivities of the WO_3_ sensors are much enhanced by mixing Ag. Responses of pure WO_3_ and Ag-WO_3_ sensors calcined at different temperatures to different concentrations of NO_2_ (0.25–20 ppm) were measured at the same operating temperature (150°C) and are shown in figure 6*b*. All the responses have the same trend that they increased with the increase in the concentration of NO_2_. Furthermore, the gas response of all the Ag-WO_3_ sensors is higher than that of the pure WO_3_ sensors at the same condition. It can also be observed that Ag-WO_3_ calcined at 500°C exhibits the highest gas response, which may be due to the good crystallization and biggest specific surface area. The response of all sensors has the biggest increasing rate in the range of 5–10 ppm NO_2_, which indicated that the sensors have an excellent performance in monitoring NO_2_ gas, especially in low concentrations.

**Figure 7.**
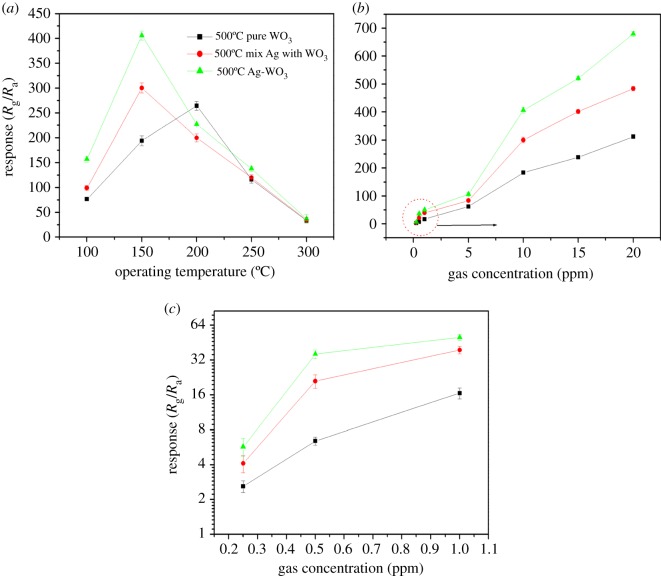
(*a*) Gas sensing response of the sensors based on pure WO_3_, mixture of Ag and WO_3_, Ag-WO_3_ calcined at 500°C to 10 ppm NO_2_. (*b,c*) Responses of pure, mixture of Ag and WO_3_ and Ag-WO_3_ sensors to different concentration NO_2_ at 150°C.

To highlight the highest performance of Ag-WO_3_ calcined at 500°C, Ag and WO_3_ components were separately synthesized by the sol-gel method and calcined at 500°C and then a control sample of the physical mixture of Ag and WO_3_ was tested for sensing NO_2_. Ag particles can be prepared by sol-gel method through Si(OC_2_H_5_)_4_, AgNO_3_ and HNO_3_ [44]. Two grams of silver particles was fully mixed with 200 g WO_3_ and its sensor performance is shown in figure 7. Physical mixture of Ag and WO_3_ has the same performance as Ag-WO_3_ and its operating working temperature is lower than pure WO_3_ sensors.

It is well known that semiconductor gas sensors are surely affected by the presence of ambient moisture [45]. When chemisorbed on material, water molecules influence the conductivity. The effect of humidity on semiconductor sensor is also related to the temperature and gas composition of the sensor. So all the experiments were measured in the same presence of ambient moisture.

The gas sensing mechanism of n-type semiconductor oxide is based on the change in resistance, which is primarily caused by the chemical adsorption and reaction of the gas on the surface of the sensing materials. When WO_3_ is exposed in the atmosphere, oxygen molecules are adsorbed on the surface, and changed into chemisorbed oxygen species (O^2−^, O^−^) by capturing free electrons from conduction band. Presence of these oxygen species is decided by the operating temperature.

The oxygen ions predominantly exist in the form of O^2−^ below 100°C, O^−^ between 100 and 300°C. Above 300°C the O^2−^ are produced and get directly incorporated into the lattice. When the operating temperature of the material is 150°C, as represented in equations (3.1)–(3.3).

3.1 $$O_{2}(\mathrm{gas})\to O_{2}(\mathrm{ads}),$$

3.2 $$O_{2}(\mathrm{ads})+e^{-}\to O_{2}^{-}(\mathrm{ads})$$

3.3 $$\;\mathrm{and}\;O_{2}^{-}+e^{-}\to 2O^{-}.$$

Depletion region is formed on the surface of WO_3_, leading to a decrease of carrier concentration and electron mobility [46]. Exposure to NO_2_ gas results in a further decrease of the carrier concentration, for the electrons of WO_3_ are captured [47], as represented in equations (3.4)–(3.6), and the depletion width further increases, which eventually decreases the conductivity of the sensor. When the Ag-WO_3_ are exposed to NO_2_, NO_2_ as a polar molecule with positive charge localizes on the nitrogen

3.4 $$NO_{2}(\mathrm{gas})+e^{-}↔{\mathrm{NO}}_{2}^{-}(\mathrm{ads}),$$

3.5 $$NO_{2}(\mathrm{gas})+e^{-}↔\mathrm{NO}(\mathrm{gas})+O^{-}(\mathrm{ads})$$

3.6 $$\;\mathrm{and}\;NO_{2}(\mathrm{gas})+O_{2}^{-}(\mathrm{ads})+2e\to {\mathrm{NO}}_{2}^{-}(\mathrm{ads})+2O^{-}(\mathrm{ads}).$$

and negative charge on the oxygen atoms, and electron interaction with the Ag will repel the negatively charged oxygen and attract the positively charged nitrogen [48]. The sensing properties of Ag-WO_3_ materials are enhanced compared with pure WO_3_ material due to the catalytic activity of Ag nanoparticles. The Ag additive serving as an active catalyst plays an important role in enhancing sensitivity, which can create more active sites [49]. Furthermore, the underneath areas of Ag particles will be less depleted by the electron flow from Ag to WO_3_, for the work function of Ag (4.26 eV) is smaller than that of WO_3_ (5.05 eV) [50].

**Figure 8.**
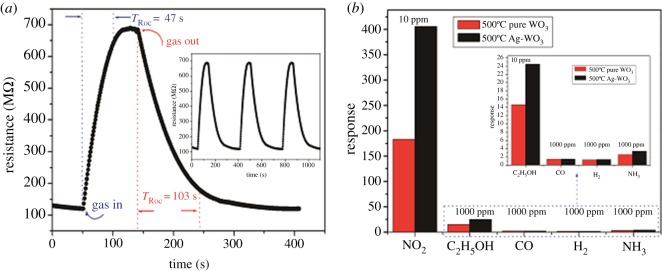
(*a*) Response and recovery characteristic of Ag-WO_3_ sensor calcined at 500°C to 250 ppb NO_2_ while cycling NO_2_ gas in and out of the measurement environment three times at 150°C. (*b*) Responses of pure WO_3_ and Ag-WO_3_ sensor calcined at 500°C to various gases at 150°C (ethanol, CO, H_2_ and NH_3_ at 1000 ppm and NO_2_ at 10 ppm). Responses: *S* = *R*_g_/*R*_a_ for oxidizing gases or *R*_a_/*R*_g_ for reducing gases.

**Figure 9.**
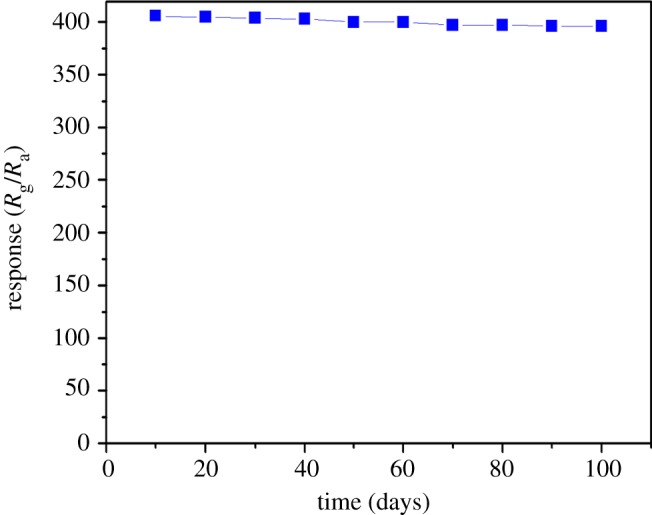
The long-term response values of an Ag-WO_3_ sensor calcined at 500°C to 10 ppm NO_2_ at 150°C.

As is known to all, response and recovery characteristics are important for estimating the performance of a sensor. The resistance changes of Ag-WO_3_ powder calcined at 500°C were repeatable for three successive measures in 250 ppb NO_2_. The response and recovery times in a single cycle were about 47 s and 103 s, respectively, as shown in figure 8*a*. Figure 8*b* shows relative responses of pure WO_3_ and Ag-WO_3_ power sensors calcined at 500°C on exposure to different gases (ethanol, CO, H_2_, and NH_3_ at 1000 ppm and NO_2_ at 10 ppb) at 150°C. The responses of the two sensors exhibited a high performance of NO_2_, while they are a little sensitive to four gases. Moreover, compared to the pure WO_3_ sensor, the Ag-WO_3_ sensor exhibited higher responses to all the testing gas, especially to NO_2_. As a result, the Ag-WO_3_ sensor can be a very promising sensor to monitor NO_2_ at relatively low temperature; both sensitivity and selectivity are taken into consideration. The long-term stability is important to ensure the accuracy of detection for gas sensors. Consequently, a test for the long-term stability of sensor calcined at 500°C to 10 ppm NO_2_ was measured for three months. As shown in figure 9, it can be observed that the sensor still showed excellent response performance to NO_2_ gas even after three months, and the response values were just floating around 400, which indicated that the sensors based on Ag-WO_3_ have enough stability to detect NO_2_ gas for a relatively long period. The sensing properties (working temperature, response to a certain NO_2_ concentration,) of several NO_2_ sensors are compared in table 2.

**Table 2.** Comparison of NO_2_ sensing performances of the current work with other reported literature.

sensing materials	operating temperature (°C)	NO_2_ concentration	response (*R*_g_/*R*_a_)	ref.
Fe-WO_3_	120	500 ppb	2.7	[51]
ZnO@Au	250	1 ppm	10.7	[52]
WO_3_	220	500 ppb	50.7	[53]
mesoporous In_2_O_3_	150	250 ppb	10.5	[54]
In_2_O_3_ nanosheet	250	50 ppm	164	[55]
In_2_O_3_/NiO	room temperature	15 ppm	3	[56]
SnS_2_	120	10 ppm	36.3	[57]
MoS_2_	200	1 ppm	5.8	[58]
ZnO	290	40 ppm	264	[59]
ZnO/CNT	150	1000 ppm	9.7	[60]
(500°C) Ag-WO_3_	150	10 ppm	408	this work

## 4. Conclusion

In summary, WO_3_ and Ag-WO_3_ (mixed solid solutions Ag with WO_3_) nanoparticles were successfully fabricated by sol-gel method. The XRD results show that the hexagonal to monoclinic phase transition takes place in the temperature range from 300°C to 500°C. The crystalline size of WO_3_ nanoparticles increases with increasing calcination temperature and decreased slightly by mixing Ag. The gas sensing properties of Ag-WO_3_ nanoparticles were measured and the experimental results exhibit that the gas sensor based on Ag-WO_3_ nanoparticle film has excellent selectivity and long-term stability to NO_2_ gas. The operating temperature and the amounts of additives play an important role in the response of the sensors. The optimum performance was obtained at 150°C for the Ag-WO_3_ sensor calcined at 500°C and can be suitable for detecting NO_2_ at relatively low operating temperature.

## Supplementary Material

N2 adsorption-desorption isotherms

## Data accessibility

This article has no additional data.
