# Supplementary material for: Effects of sintering temperature on sensing properties of WO3 and Ag-WO3 electrode for NO2 sensor
Source: R Soc Open Sci. 2018 Oct 31;5(10):171691. doi: 10.1098/rsos.171691 (PMC6227965; doi:10.1098/rsos.171691)
Supplement: N2 adsorption-desorption isotherms [file rsos171691supp1.docx]

Electronic Supplementary Materials

Effects of sintering temperature on sensing properties of WO_3_ and Ag-WO_3_ electrode for NO_2_ sensor

Rui Lu^1^, Xiaoling Zhong*^1^, Shiguang Shang^2^, Shan Wang^3^ and Manling Tang^1^

^1^College of Information Science &Technology, Chengdu University of Technology, Chengdu 610059, People’s Republic of China

^2^School of Electronic Engineering, Xi'an University of Posts and Telecommunications, Xi'an 710121, People’s Republic of China

^3^Faculty of Automation and Information Engineering, Xi'an University of Technology, Xi'an 710048, People’s Republic of China

Nitrogen adsorption-desorption isotherms and pore diameter distribution curves


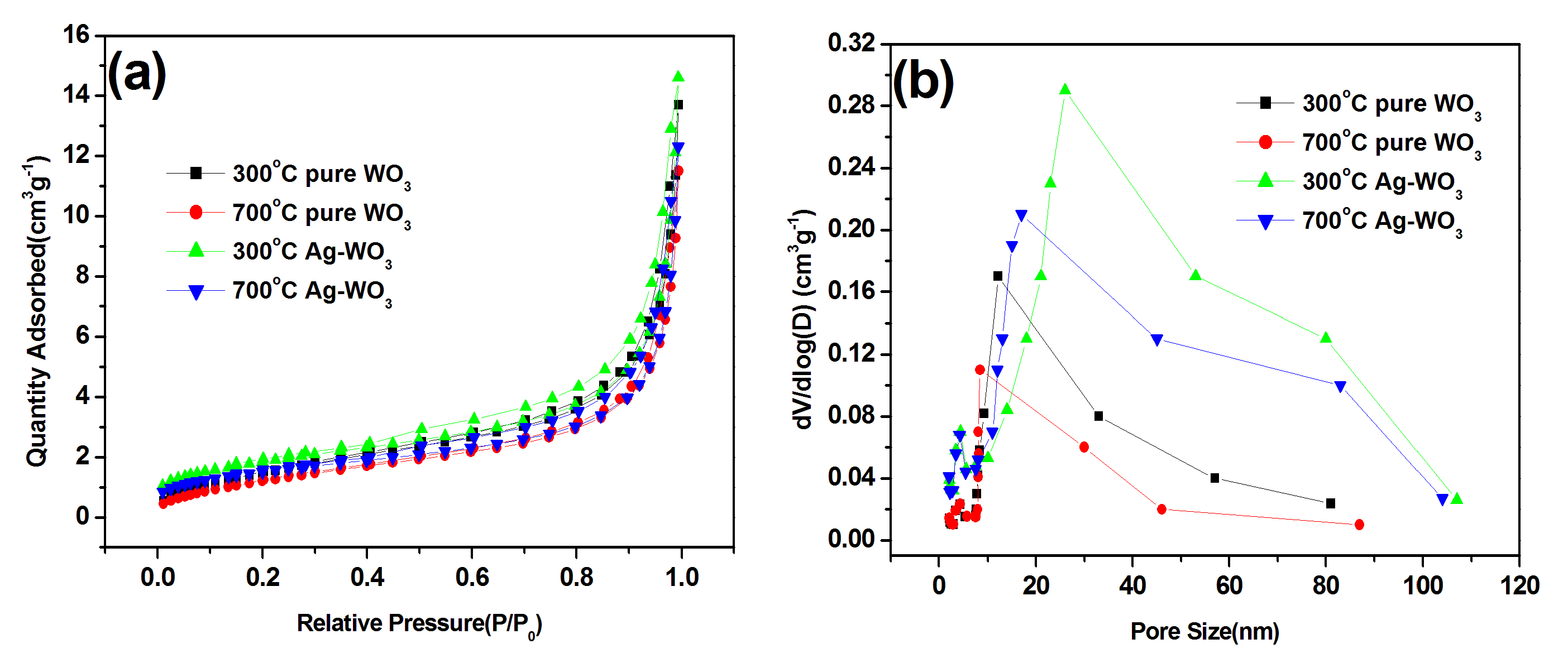


**Figure S1.** (a) Nitrogen adsorption-desorption isotherms and (b) pore diameter distribution curves of WO_3_ and Ag-WO_3_ calcined at 300^o^C.and 700^o^C
